# Alterations of theta power and synchrony during encoding in young adult binge drinkers: Subsequent memory effects associated with retrieval after 48 h and 6 months

**DOI:** 10.3389/fpsyg.2022.1061016

**Published:** 2022-12-15

**Authors:** Siyuan Huang, David R. White, Ksenija Marinkovic

**Affiliations:** ^1^Spatio-Temporal Brain Imaging Laboratory, Department of Psychology, San Diego State University, San Diego, CA, United States; ^2^Department of Radiology, University of California, San Diego, San Diego CA, United States

**Keywords:** binge drinking, EEG, subsequent memory effects, memory encoding, theta power, phase-locking synchrony

## Abstract

**Introduction:**

Young emerging adults commonly engage in binge drinking which is associated with a range of neurocognitive deficits, including memory impairments. However, evidence on neural oscillations mediating episodic memory in this population is lacking. To address this gap, we recorded theta oscillatory activity in young binge (BDs) and light drinkers (LDs) during memory encoding and analyzed it prospectively as a function of subsequent retrieval. Theta underlies successful encoding of novel items in memory through corticolimbic integration. Subsequent memory effects (SMEs) are reflected in stronger theta activity during encoding of the items that are later remembered compared to those that are later forgotten.

**Methods:**

In the present study, 23 BDs (age: 23.3 ± 3.3) and 24 LDs (age: 23.4 ± 3.3) rated emotionally evocative images with negative, positive, and neutral themes during implicit encoding. They performed a recognition memory task on two follow-up occasions after a short (48 h), and long retention delay (6 months). Electroencephalography (EEG) signal was recorded during the encoding session and analyzed in time-frequency domain with Morlet wavelets in theta band (4–7 Hz). To evaluate SMEs, the event-related theta oscillations acquired during encoding were analyzed based on recognition outcomes after the two retention intervals.

**Results:**

The BD and LD groups did not differ on recognition memory. However, BDs showed attenuated event-related theta power during encoding of images that were successfully retained after 6 months compared to LDs. In addition, theta synchronous activity between frontal and left posterior regions during encoding successfully predicted recognition of the images after both retention delays in LDs but not in BDs. These SMEs on theta power and synchrony correlated negatively with high-intensity drinking in the previous 6 months. No differences between men and women were observed for any analysis.

**Discussion:**

It has been well established that long-range neural synchrony between cortical and limbic nodes underlies successful memory encoding and retention which, in turn, depends on neural excitation/inhibition (E/I) balance. Given that binge drinking is associated with E/I dysregulation, the observed SME deficiencies are consistent with other evidence of neural hyperexcitability in BDs, and may be indicative of increased risk of developing alcohol use disorders.

## Introduction

Memory plays a fundamental role in connecting the past and the present while providing a conceptual framework needed to predict the future and to manage ongoing activities as they unfold in real time (Tulving, 2000; Hawkins and Blakeslee, 2007). Remembering a stimulus relies on successful encoding, consolidation, and retrieval of information (Tulving, 1972). A typical memory-probing paradigm comprises two experimental occasions: during an encoding session, participants are presented with a list of items. During the subsequent recognition session, the participants are asked to classify each item as old (previously encountered) or new. Behavioral experiments have shown that high rates of recognition with or without recollecting details of the encoding episode persist for long periods of time (Gardiner and Java, 1991; Dewhurst et al., 2009; Erk et al., 2010).

EEG-based methods have been used extensively to study the neural underpinnings of memory processes, and have provided insight into their dynamics with an emphasis on theta oscillations (Friedman and Johnson, 2000; Jacobs et al., 2006; Werkle-Bergner et al., 2006; Bäuml et al., 2008). Intracranial EEG (iEEG) human studies have established that hippocampal theta underlies successful encoding through interactions with cortical and limbic areas, confirming that the distributed oscillatory neural activity enables coherent integration across multiple brain areas (Lega et al., 2012; Lin et al., 2017; Zheng et al., 2019). Evidence obtained during word encoding indicates that increased frontal and temporal theta power predicts better subsequent word recognition (Sederberg et al., 2003). Similarly, both magnetoencephalography (MEG) and scalp EEG studies have reported greater theta power during encoding of the photos that were later recognized compared to those that were later forgotten (Klimesch et al., 1997; Summerfield and Mangels, 2005; Hanslmayr et al., 2009). This line of evidence suggests that theta oscillations mediate the long-range synchronous coactivation of the hippocampus and the cortex, and the long-range cortico-cortical connections during encoding of new information (Wang et al., 2005; Hasselmo and Stern, 2014; Hsieh and Ranganath, 2014). Memory consolidation, or the establishment of stable memories over time, relies on the hippocampus to guide reorganization of the information encoded in distributed cortical regions (Squire et al., 2015). Greater activity during encoding of the subsequently remembered, compared to the subsequently forgotten items, has been termed the subsequent memory effect (SME), also known as difference due to memory [Dm] effect (Sanquist et al., 1980; Paller and Wagner, 2002). Studies using the SME paradigm have reported higher theta power during encoding of the items that were later recalled, highlighting its importance for successful encoding (Klimesch et al., 1997; Sederberg et al., 2003; Osipova et al., 2006).

It has been well established that acute alcohol intoxication disrupts memory encoding (Weissenborn and Duka, 2003; White, 2003; Mintzer, 2007; Doss et al., 2018). However, exceedingly few studies have investigated alcohol-induced changes of oscillatory activity during memory tasks. Krause et al. (2002) reported a decrease in event-related theta during both encoding and subsequent retrieval of auditory stimuli during acute alcohol administration, which is consistent with alcohol-induced increase of neural inhibition (Kumar et al., 2009; Doss et al., 2018; Correas et al., 2021). Even though it is known that excessive alcohol consumption has detrimental impact on memory (Oscar-Berman et al., 2014; Fama et al., 2021), to our knowledge, there is currently no available evidence on oscillatory dynamics underlying episodic memory impairments associated with alcohol use disorder (AUD). In contrast, multiple studies have examined other cognitive functions such as inhibitory control and attention, and have reported alterations in theta oscillations following chronic excessive alcohol exposure (Kamarajan et al., 2004; Porjesz et al., 2005; Rangaswamy and Porjesz, 2014). The anomalies in event-related theta oscillations during cognitive tasks have also been observed in the offspring of individuals diagnosed with alcohol use disorder (AUD) (Kamarajan et al., 2006; Rangaswamy et al., 2007), suggesting that event-related theta oscillations could serve as an endophenotype for susceptibility to alcohol addiction (Porjesz et al., 2005; Porjesz and Rangaswamy, 2007).

Heavy episodic drinking, also termed binge drinking, is a pattern of alcohol consumption that elevates one’s blood alcohol concentration to or above the legal intoxication level (0.08 g/dl, National Institute of Alcohol, Abuse and Alcoholism, 2022). It is commonly practiced among young, emerging adults, and is associated with neurocognitive deficits, including low academic performance (Miller et al., 2007; Pascarella et al., 2007; Petit et al., 2014; Lannoy et al., 2019). Consistent with these reports, some studies have confirmed a linkage between binge drinking and poor performance on both verbal (Sneider et al., 2012; Mota et al., 2013; Carbia et al., 2018) and visual memory tasks (Weissenborn and Duka, 2003; Hartley et al., 2004), as well as face-name encoding deficits (Folgueira-Ares et al., 2017). However, the changes in oscillatory brain dynamics characterizing memory formation in healthy, young adults with a history of binge drinking have yet to be investigated.

To address this gap, the present study examined theta-based indices of memory encoding that predict recognition outcomes as a function of habitual binge drinking. During an implicit encoding session, young adults with or without a history of binge drinking were presented with pictures depicting a range of emotional scenes and were asked to rate how they felt about each picture (Huang et al., 2018). Subsequently, the strength of their memory trace was probed with recognition tasks conducted with delays of 48 h (hrs) and 6 months (mos) respectively. To characterize SMEs, event-related theta oscillations were examined during encoding as a function of recognition outcomes recorded after these two intervals and compared between the two groups. Furthermore, given the importance of neural synchronization for memory formation (Clouter et al., 2017; Eichenbaum, 2017), we investigated the strength of theta co-oscillations during encoding as a function of the recognition delay. We hypothesized that the recognition rates, event-related theta power during encoding of the pictures, as well as theta co-oscillations, would be attenuated in individuals who engage in binge drinking as compared to the demographically matched moderate social drinkers, particularly for the 6-mos retention interval.

## Materials and methods

### Participants

Sixty-eight young, healthy adults (average age 23.3 ± 3.3 yrs., age range: 18–30 yrs., 34 women) were recruited from the local community through flyers and ads. They were all right-handed and reported no illegal drug, cannabis, or tobacco use at least 1 month prior to the study, no history of seizures, brain injury, neurological or neuropsychiatric disorders, no vision, hearing, or learning problems, and no medication use at the time of the study. This information was obtained in an initial online screening survey, and was queried in greater detail in a follow-up phone interview. Based on questionnaires querying their current and recent drinking patterns, they were assigned to Binge Drinking (BD) and Light Drinking (LD) groups (Table 1). The BD group comprised 34 participants (17 women) who reported at least five binge episodes in the past 6 months and at least one binge episode in the previous month, with 13.2 ± 8.9 binge episodes on average. A binge episode was defined as consuming at least 6 (men) or 5 (women) drinks within a two-hour time span. This criterion was adopted based on the evidence suggesting that this level of drinking is likely to result in blood alcohol concentration (BAC) of 0.08% or above (Lange and Voas, 2001). The remaining 34 participants (17 women) who reported no more than one binge episode in the past 6 months were assigned to a LD group. No abstainers were recruited as all LDs reported consuming at least 1 drink per week on average. The two groups were matched on age, sex, education, ethnicity/race, and family history of AUD (Table 1). They took part in an encoding session (ENCODE), followed by a recognition session scheduled 48 h later. In addition, 19 BDs and 25 LDs participated in a third session probing recognition after a 6-mos long retention interval.

**Table 1:** Participant characteristics [Mean ± SD or *n* (%)] for the BD and LD groups assessed at enrollment and at a 6-month retention interval.

	At enrollment			After a 6-month retention interval			Main effects of Time (change after 6 months)	
	BD (*n* = 34)	LD (*n* = 34)	*p*	BD (*n* = 19)	LD (*n* = 25)	*p*	BD (*n* = 19) *p*	LD (*n* = 25) *p*
**Demographics**								
% Women	50%	50%	1.0^a^	52.6%	60%	0.63^a^		
% White/non-Hispanic	67.6%	70.6%	0.79^a^	63.2%	68.0%	0.74^a^		
Age	23.3 ± 3.3	23.4 ± 3.3	0.81	23.8 ± 3.5	23.3 ± 3.2	0.69		
Age range	18–30	18–29		18–30	18–29			
% Fam. hist. of AUD	50.0%	41.2%	.47^a^	42.1%	48.0%	0.70^a^		
Education years	15.7 ± 2.0	16.3 ± 2.3	0.18	15.4 ± 1.9	16.3 ± 2.4	0.28		
Undergraduate GPA	3.15 ± 0.46	3.46 ± 0.37	0**.004**	3.02 ± 0.51	3.54 ± 0.24	**0.001**		
BMI	24.92 ± 3.94	23.19 ± 3.22	0.06	25.71 ± 4.64	23.11 ± 2.56	**0.04**		
**Drinking-related variables**								
Age of drinking onset	16.0 ± 1.4	18.5 ± 2.0	**< 0.001**					
Alcoholism sympt. (SMAST)	3.51 ± 3.60	0.53 ± 0.86	**<0.001**	2.63 ± 3.35	0.24 ± 0.52	**<0.001**	0.62	0.16
In the past 6 months:								
Drinks per week	17.3 ± 8.4	3.0 ± 1.9	**<0.001**	14.1 ± 11.1	3.3 ± 2.7	**<0.001**	0.15	0.29
Intoxications per month	5.6 ± 4.5	1.9 ± 1.7	**<0.001**	6.4 ± 6.5	0.8 ± 1.0	**<0.001**	0.56	. 07↓
Binge episodes	13.2 ± 8.9	0.09 ± 0.3	**<0.001**	7.6 ± 5.6	0.2 ± 0.6	**<0.001**	0.41	0.10
Alcohol-induced blackouts	4.4 ± 3.5	0.03 ± 0.2	**<0.001**	3.3 ± 3.4	0	**<0.001**	0.28	0.33
Max. Drinks per occasion	12.7 ± 5.8	4.7 ± 2.1	**<0.001**	12.0 ± 7.7	4.1 ± 2.3	**<0.001**	0.39	0.72
Drinking motives (DMQ-R)								
Enhancement	2.21 ± 0.46	1.77 ± 0.50	**0.001**					
Social	2.52 ± 0.44	2.08 ± 0.54	**0.001**					
Conformity	1.41 ± 0.48	1.35 ± 0.41	0.66					
Coping	1.66 ± 0.58	1.24 ± 0.32	**0.001**					
**Internalizing variables**								
Anxiety (GAD-7)	4.03 ± 5.11	2.12 ± 2.55	0.22	3.11 ± 2.99	1.79 ± 2.11	0.19	0.71	0.41
Depression (PHQ-9)	4.39 ± 4.80	1.88 ± 1.62	0.09	3.06 ± 2.44	1.96 ± 1.99	0.12	0.47	0.50
**Cognitive battery**								
NIH-Toolbox Cognitive Tests								
Working Memory/List Sorting	107.66 ± 13.01	104.01 ± 13.26	0.35					
Cognitive Flexibility/DCCS	104.74 ± 9.38	108.39 ± 8.05	0.10					
Processing Speed/Pattern Comparison	129.14 ± 14.35	132.71 ± 18.40	0.24					
Episodic Memory/PSM	113.89 ± 15.08	115.08 ± 12.72	0.56					

Fam. hist. of AUD, family history of AUD; sympt., symptoms; SMAST, Self-Administered Short Michigan Alcoholism Screening Test; Max., maximum number of; DMQ-R, Drinking Motive Questionnaire Revised Short Form; GAD-7, 7-item anxiety scale; PHQ-9, 9-item Patient Health Questionnaire; DCCS, Dimensional Change Card Sort; PSM, Picture Sequence Memory. ^a^Chi-square test. ↓Decrease from enrollment to a 6-month retention interval. Bold values refer to p values that reach the significance level (*p* < 0.05).

### Procedure

Participants completed a battery of questionnaires including handedness (Oldfield, 1971), medical history, drinking habits including the frequency and quantity of alcohol consumption (modified from Cahalan et al., 1969), the severity of alcoholism-related symptoms (Self-Administered Short Michigan Alcoholism Screening Test, SMAST; Selzer et al., 1975), and motives for engaging in alcohol use (Drinking Motive Questionnaire Revised Short Form, DMQ-R SF; Kuntsche and Kuntsche, 2009). A modified version of the Family History Assessment Module (FHAM; Rice et al., 1995) was administered to assess family history of AUD. Family history positive (FHP) participants were those who reported at least one first-degree and one first-or second-degree relative, or at least three second-degree relatives. Prospective participants with a maternal history of alcohol misuse were excluded from the study to avoid possible fetal alcohol exposure confounds. Family history negative (FHN) participants reported no first-or second-degree biological relatives with problem drinking or AUD. A subset of participants (6 BDs and 4 LDs) did not meet the criteria for either negative or positive family history. In addition, participants completed questionnaires measuring depression (9-item Patient Health Questionnaire, PHQ-9; Kroenke and Spitzer, 2002) and anxiety (7-item anxiety scale, GAD-7; Spitzer et al., 2006). The participants also completed the NIH-Toolbox Cognitive Battery comprising the List Sorting Working Memory Test to assess working memory capacity, Dimensional Change Card Sort (DCCS) Test to assess cognitive flexibility, Pattern Comparison Processing Speed Test to measure processing speed, and Picture Sequence Memory (PSM) Test which probed episodic memory (Gershon et al., 2013).

Participants came to the lab on three occasions. The first (ENCODE) and the second (48-h) experimental sessions were scheduled exactly 48 h apart and the third session followed after 6 mos. The participants were asked to refrain from consuming any alcohol at least 48 h prior to each experimental session. Upon arrival at the lab, they provided a urine sample and all tested negative on a 12-panel multidrug test (American Screening Corporation, United States). In the ENCODE session, participants completed an emotional rating task the results of which have been reported in a separate manuscript (Huang et al., 2018). This rating task served as an implicit encoding of the pictorial stimuli while the EEG signals were recorded from the electrodes placed on the scalp. In the two subsequent sessions participants took part in recognition tasks probing their recent (48-h) and remote (6-mos) memory of these pictures. Participants were monetarily compensated for taking part in the study.

### Material

**Figure 1:**
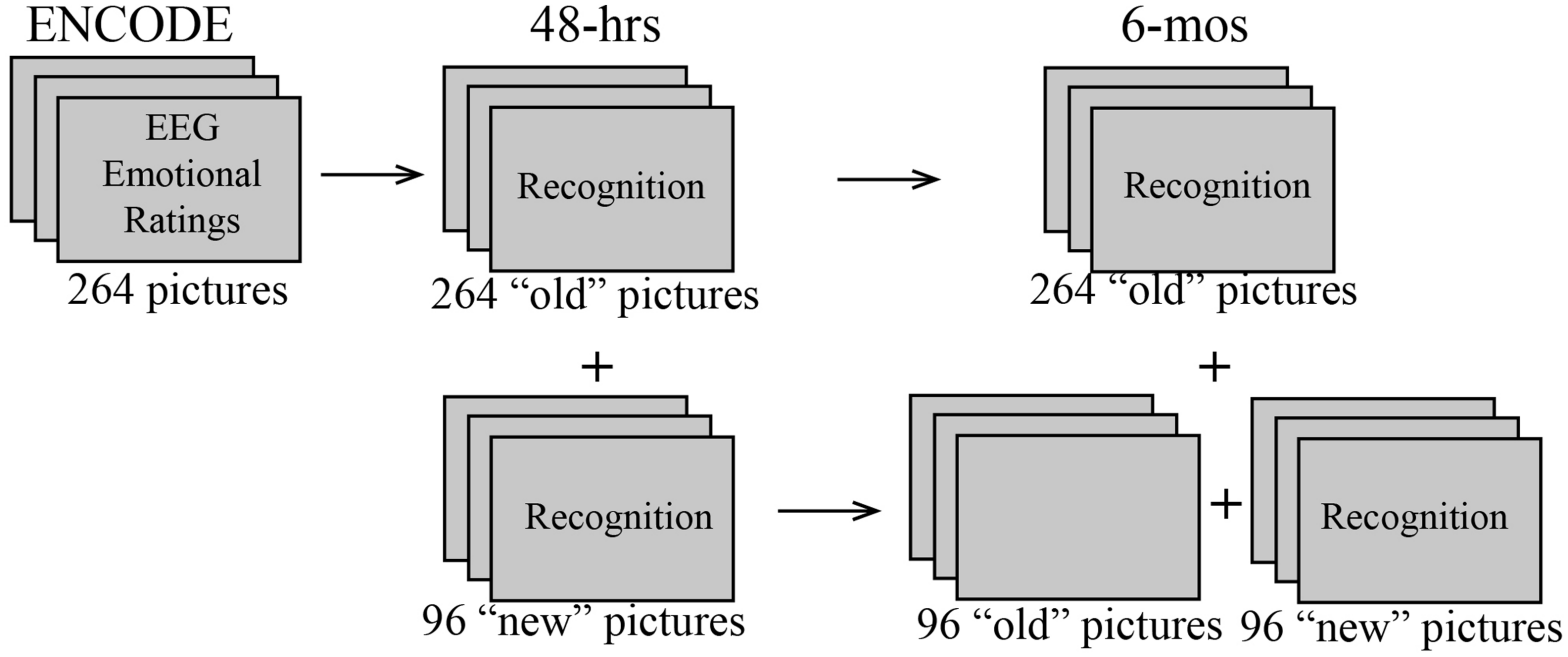
Schematic diagram of the study design showing picture sets that were used in the ENCODE, and 48-h and 6-mos recognition sessions. To examine subsequent memory effects (SME), EEG data from the ENCODE session were analyzed as a function of recognition outcomes observed in the 48-h and 6-mos sessions. In the 6-mos session, the unmarked pictures were used as lures but were not analyzed for recognition accuracy.

During all three sessions participants were presented with color pictures depicting scenes with negative, neutral, or positive valence, which were selected mostly from the International Affective Picture System (IAPS; Lang et al., 2008). In the ENCODE session, 264 pictures were included in the emotional rating task. EEG analyses were carried out only on these initial 264 pictures. They were used as “old” (previously seen) items in both 48-h and 6-mos recognition sessions (Figure 1). In each recognition session, additional 96 pictures were presented as “new,” not previously seen items. Importantly, the “new” pictures included in the 48-h recognition session were used as lures in the 6-mos recognition session, but were excluded from the behavioral analysis of that session. The “old” and “new” sets were randomly selected from a larger picture set and were equated in valence, arousal ratings, and the presence of human faces. The stimulus set contained an equal number of pictures with positive, neutral, and negative emotional valence. For more details on picture selection and characteristics please see a related article (Huang et al., 2018).

### Task descriptions

In all three experiments, each trial started with a fixation cross shown on the screen for 1,000 ± 250 ms, followed by a picture presented for 1,000 ms in the center of a 24-inch color monitor subtending a visual angle of 6.0° × 4.5° at a viewing distance of 180 cm. Pictures were presented in a randomized order in eight blocks. After the offset of each picture, a scale was shown on the screen for 2,700 ms. In the emotional rating task during ENCODE, participants were instructed to judge how each image made them feel on a 9-point visual Likert scale ranging from 1 (very negative) to 9 (very positive) by using a joystick. In the subsequent 48-h recognition session, participants were asked to judge whether they had seen each picture during ENCODE (remembered/old) or not (new) and to indicate the confidence level on a 4-point rating scale (1 = high-confidence new, 2 = low-confidence new, 3 = low-confidence old, 4 = high-confidence old) with a joystick. Similarly, in the 6-mos delay recognition session, they judged whether they had seen the picture before and the confidence level on the same scale. The experiment was conducted with a PC using the Presentation software (Neurobehavioral Systems Inc.). Before each recording, participants practiced the task with additional 20 images that were excluded from the actual experiment.

### EEG recording

EEG signals were recorded from a 64-channel actiCap DC Brain Vision system (Brain Products GmbH, Germany) and were continuously sampled at 500 Hz. The nose served as the reference and an electrode attached to the forehead as ground. Eyeblinks and eye movements were monitored with bipolarly referred electrodes attached above and below the left eye. The electrode impedance was maintained below 5 kΩ.

EEG data were analyzed with customized MATLAB (Mathworks, Natick, MA) routines that incorporated modules from publicly available packages including Fieldtrip (Oostenveld et al., 2011) and EEGLAB (Delorme and Makeig, 2004). The continuous EEG data were bandpass filtered from 0.1 to 100 Hz, and segmented into epochs extending from-300 to 1,000 ms relative to each stimulus onset. A 300 ms long padding was added to each end of the epoch to account for edge artifacts during the wavelet analysis. The data were carefully inspected and the trials that contained obvious artifacts were rejected. An independent component analysis was used to detect and remove artifacts caused by eyeblinks and heartbeat (Delorme and Makeig, 2004). Complex power spectra were calculated for each trial using Morlet wavelets (Oostenveld et al., 2011) in 1 Hz increments with 500 ms cycle length and 2–4 cycles across all frequencies in theta range (4–7 Hz) (Marinkovic et al., 2012). Theta band wavelet results were visually inspected and any additional trials that were contaminated by artifacts were rejected. Analysis of the raw theta power in the baseline showed no group or condition differences, indicating that any observed stimulus-related differences were due to event-related changes in theta power and not to the overall differences in the baseline. Event-related theta power was averaged across theta band frequencies (4–7 Hz) and across trials for each condition and expressed as percent signal change from the average theta power during the-300 to 0 ms prestimulus baseline. To examine signal distribution across the scalp, event-related theta indices were averaged into five electrode clusters representing the frontal (comprising AFz, AF3, AF4, Fz, F1, F2, F3, F4, F5, F6 electrodes), central (FCz, FC1, FC2, FC3, FC4, FC5, FC6, Cz, C1, C2, C3, C4, C5, C6), parietal (CPz, CP1, CP2, CP3, CP4, CP5, CP6, Pz, P1, P2, P3, P4, P5, P6), left temporal (FT7, T7, TP7, TP9, P7), and right temporal (FT8, T8, TP8, TP10, P8) montage areas (López-Caneda et al., 2013, 2014). Co-oscillations between the midline frontal (Fz) and the electrodes positioned over the left and right temporal areas were estimated by calculating phase-locking values (PLV) which reflect the consistency of phase differences in theta frequency band irrespective of the amplitudes of the neural activity (Lachaux et al., 1999; Tallon-Baudry and Bertrand, 1999; Beaton et al., 2018; Correas et al., 2019; Marinkovic et al., 2019). PLVs were expressed as percent signal change from the baseline. All event-related changes were quantified by analyzing a time window capturing the peak activity.

### Statistical analyses

The subsequent memory effects (SMEs) for the encoding EEG signals were analyzed as a function of retrieval after two retention delays. Specifically, the EEG trials recorded during the ENCODE session were divided into the trials that were later remembered with high-confidence, and those that were later forgotten, as indicated by recognition performance after 48 h and 6 mos, respectively. Event-related theta power and PLV indices were analyzed with mixed-design analyses of variance (ANOVAs) with Group (BD vs. LD) as a between-subject factor and SME (Later Remembered and Later Forgotten) as a within-subject factor.

To examine the behavioral indices of changes in memory retrieval as a function of delay, mixed-design ANOVAs with Group (BD vs. LD) and Delay (48-h and 6-mos) were carried out on the *d-prime* (*d*’) derivation based on recognition with high (H-*d’*) and low (L-*d’*) confidence. D′ was calculated from hit rate (HIT) and false-alarm rate (FA) using the formula *d*’ = Z_HIT_ – Z_FA_ where Z represents a transformation of the two distributions allowing for comparison of measures with different ranges of absolute values (Macmillan and Creelman, 1990). Trials were aggregated across all emotional categories to ensure optimal power for the EEG analyses. Moreover, the Emotion × SME interaction effects on behavioral HIT and FA rates were comparable for BD and LD groups, all *p*s > 0.12. The factor of Sex was included initially in an overall analysis model for both the EEG and behavioral data. However, there were no main effects or interactions including Sex, so it was omitted from the reported analyses. Moreover, the factor of Brain Region (frontal, central, parietal, left temporal, right temporal) was included in an initial analysis of the theta activity, but no effects on SMEs were found, *p*s > 0.10 (Supplementary Table S1). However, the analyses focused on the frontal electrodes where the memory modulations of theta activity appeared most prominent. To estimate fronto-temporal interactions, PLVs were primarily calculated for the Fz-C5 and Fz-C6 electrode pairs, which permitted laterality comparisons. PLV values for all other calculations between Fz and other lateral electrodes are available in the Supplementary Table S2.

To investigate the changes in participant self-reports across the 6-month time period, the questionnaire scores were analyzed with mixed model ANOVAs with Group as a between-group factor and Delay (at enrollment vs. 6 months later) as a within-subject factor. Group differences in dispositional variables were evaluated with the Mann–Whitney *U* tests for independent sample comparisons given that many variables violated the assumption of normal distribution. A Chi-square test was used for categorical variables such as sex and race/ethnicity. Spearman’s rank correlation analyses were performed on the intensity of drinking behaviors/symptoms and the strength of the EEG-based brain activity. Of note, one BD participant who reported a number of binge episodes beyond three standard deviations of the mean was excluded from all correlation analyses.

## Results

### Drinking-related variables, personality characteristics, and cognitive functions

As expected, group differences were observed on all variables associated with alcohol consumption (Table 1), as BDs reported consuming more alcohol, engaging in more binge episodes, and experiencing more blackouts and other alcohol-related consequences than LDs. However, the BD and LD groups did not differ on NIH-Toolbox tests of cognitive functions including working and episodic memory. A follow-up after 6 months confirmed all group differences in drinking habits.

### Recognition performance

**Figure 2:**
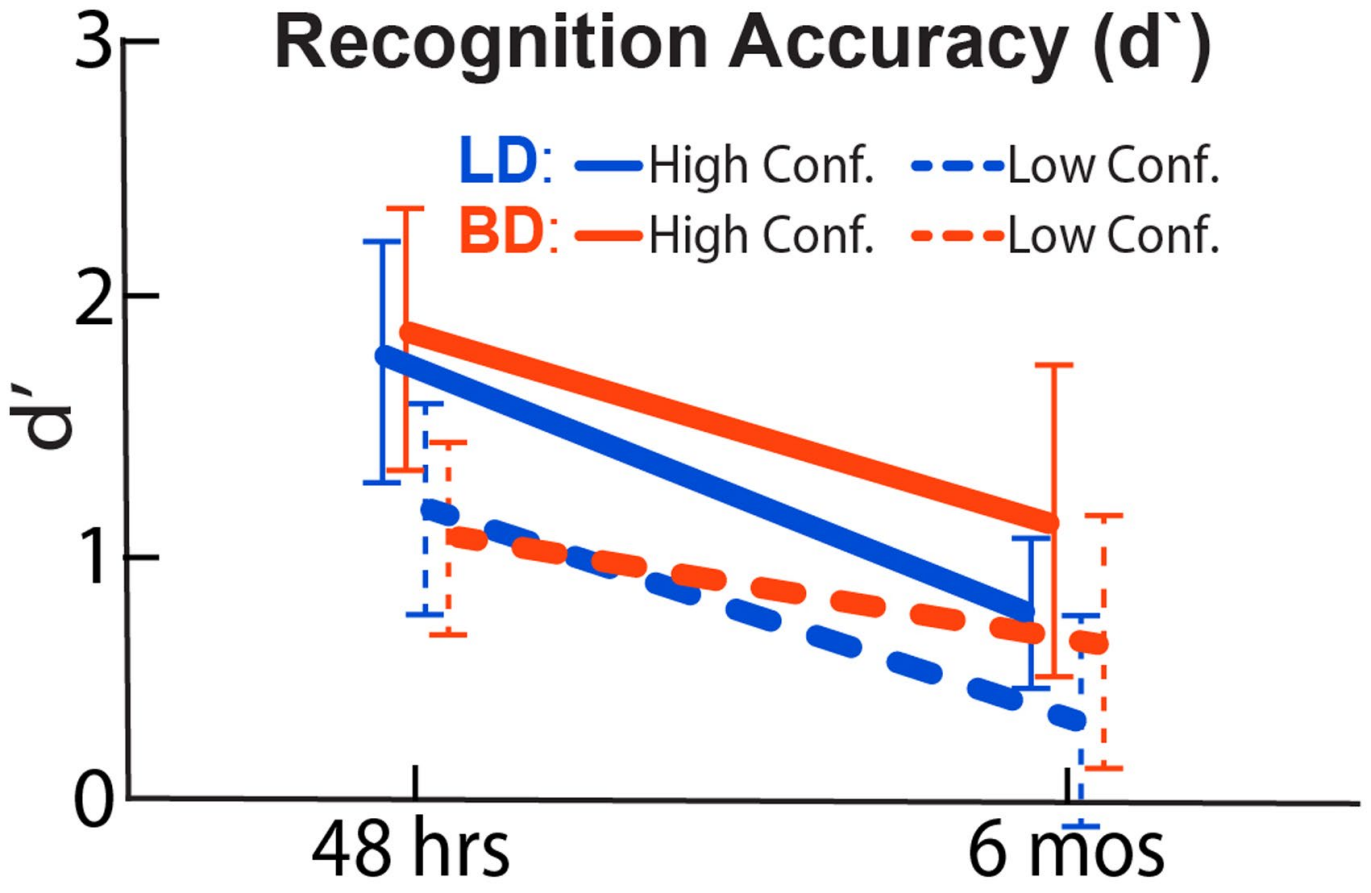
Recognition accuracy expressed as a standardized, d-prime (d’) index calculated from the hit and false-alarm rates. BD and LD groups did not differ at either 48-h or 6-mos delay. As expected, lower recognition accuracy was found after a 6-mos retention delay.

Figure 2 displays d’ for both groups based on high-confidence (H-d’) or low-confidence judgments (L-d’) during the recognition task. As expected, participants showed higher overall H-d’ after a 48-h, compared to a 6-mos delay, *F*(1, 42) = 184.43, *p* < 0.001, indicating a decrease in H-d’ across time. There was no main effect of Group, *F*(1, 42) = 0.42, *p* = 0.52, or an interaction effect of Group × Delay on H-d’, *F*(1, 42) = 0.59, *p* = 0.45. Similarly, while a reduction of L-d’ was observed after a 6-mos relative to a 48-h delay, *F*(1, 42) = 116.58, *p* < 0.001, there was no group difference, *F*(1, 42) = 0.11, *p* = 0.74, and no Group × Delay interaction, *F*(1, 42) = 0.11, *p* = 0.74.

### Subsequent memory effects: ENCODE event-related theta as a function of recognition outcomes after 48-h and 6-mos retention delays

#### 48-h delay

**Figure 3:**
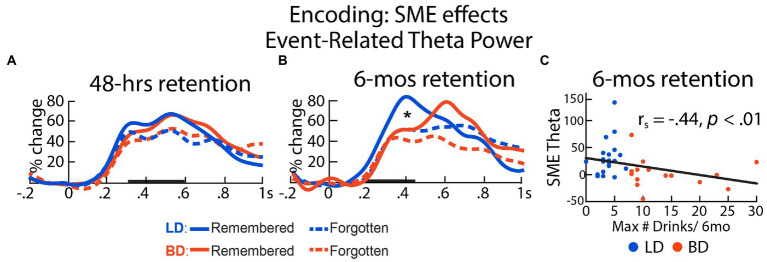
Event-related theta power during initial encoding associated with later remembered vs. forgotten pictures for LD and BD groups as a function of recognition **(A)** after 48 h and **(B)** after 6 mos. Event-related theta power is expressed as percent signal change from the baseline. **(C)** SME-related theta power difference (remembered – forgotten after 6 mos) correlated negatively with self-reported maximum number of drinks consumed on a single occasion in the past 6 months. *p ≤ 0.05. Bolded bars on the *x*-axis mark the time windows of interest.

We examined theta acquired during the ENCODE session by averaging trials that were remembered with high confidence vs. those that were forgotten after a 48-h delay. One participant in each group was excluded from the analysis due to insufficient number of trials (*n* < 15) in either condition. On average, 128 trials that were later remembered and 73 trials that were later forgotten remained in the EEG analysis for the 48-h delay. As shown in Figure 3A, a main effect of SME on encoding theta was observed within a latency interval of 300–600 ms, *F*(1, 64) = 17.50, *p* < 0.001, with higher theta power evoked by the later remembered than by the later forgotten pictures. There was no effect of Group on the overall theta power, *F*(1, 64) = 0.001, *p* = 0.97, nor on the theta power associated with recognition success, expressed as SME-related theta power difference between later remembered and forgotten, *t*(64) = 0.75, *p* = 0.45. SME-related theta power difference did not correlate with drinking/dispositional variables, all *p*s > 0.17.

#### 6-mos delay

For the analysis of ENCODE theta based on SMEs after a 6-mos long retention interval, two BDs and five LDs were excluded from the analysis due to insufficient trials, resulting in 43 remembered and 153 forgotten trials on average. A significant interaction between Group and SME within a 200–450 ms latency window was found, *F*(1, 35) = 9.08, *p* = 0.005. During encoding, only the LD group showed greater theta on the trials that were recognized vs. those that were forgotten 6-mos later, *t*(19) = 4.11, *p* < 0.001. In contrast, no SME on theta was observed in the BD group after a 6-mos delay, *t*(16) = 0.15, *p* = 0.88. SME-modulated theta power difference (later remembered – later forgotten) correlated negatively with the number of self-reported binge episodes in the past 6 months, *r*s = −0.47, *p* = 0.004, the maximum number of drinks imbibed on a single occasion in the past 6 months, *r*s = −0.44, *p* = 0.007, alcohol-induced blackout in the past 6 months, *r*s = −0.38, *p* = 0.02, and strength of social drinking motives, *r*s = −0.35, *p* = 0.036, all surviving the Benjamini-Hochberg FDR correction (Figure 3B). In a later time window (500–700 ms), SME modulated the event-related theta power, *F*(1, 35) = 5.28, *p* = 0.028, reflected by higher theta responses to the later remembered compared to the later forgotten items. However, there was no main effect of Group, *F*(1, 35) = 1.73, *p* = 0.20, or a Group × SME interaction, *F*(1, 35) < 0.001, *p* = 0.98, within this time window. The SME-modulated theta difference did not correlate with any drinking/dispositional characteristics at this latency, *p*s > 0.13. Further, we observed that the standard deviations of the SME theta power distribution in the later 500-700 ms time window (later-remembered *SD* = 0.54; later-forgotten *SD* = 0.29) appeared to be greater than that in the early 200-450 ms time window (later-remembered *SD* = 0.39; later-forgotten *SD* = 0.23). It is worth noting that none of the individual theta values exceeded three standard deviations above the group mean. The absence of group differences could be additionally attributed to the relatively small number of participants who completed the memory recognition task after a 6-mos retention interval.

### Neural synchrony during encoding: SMEs after 48-h and 6-mos retention delays

#### 48-h delay

**Figure 4:**
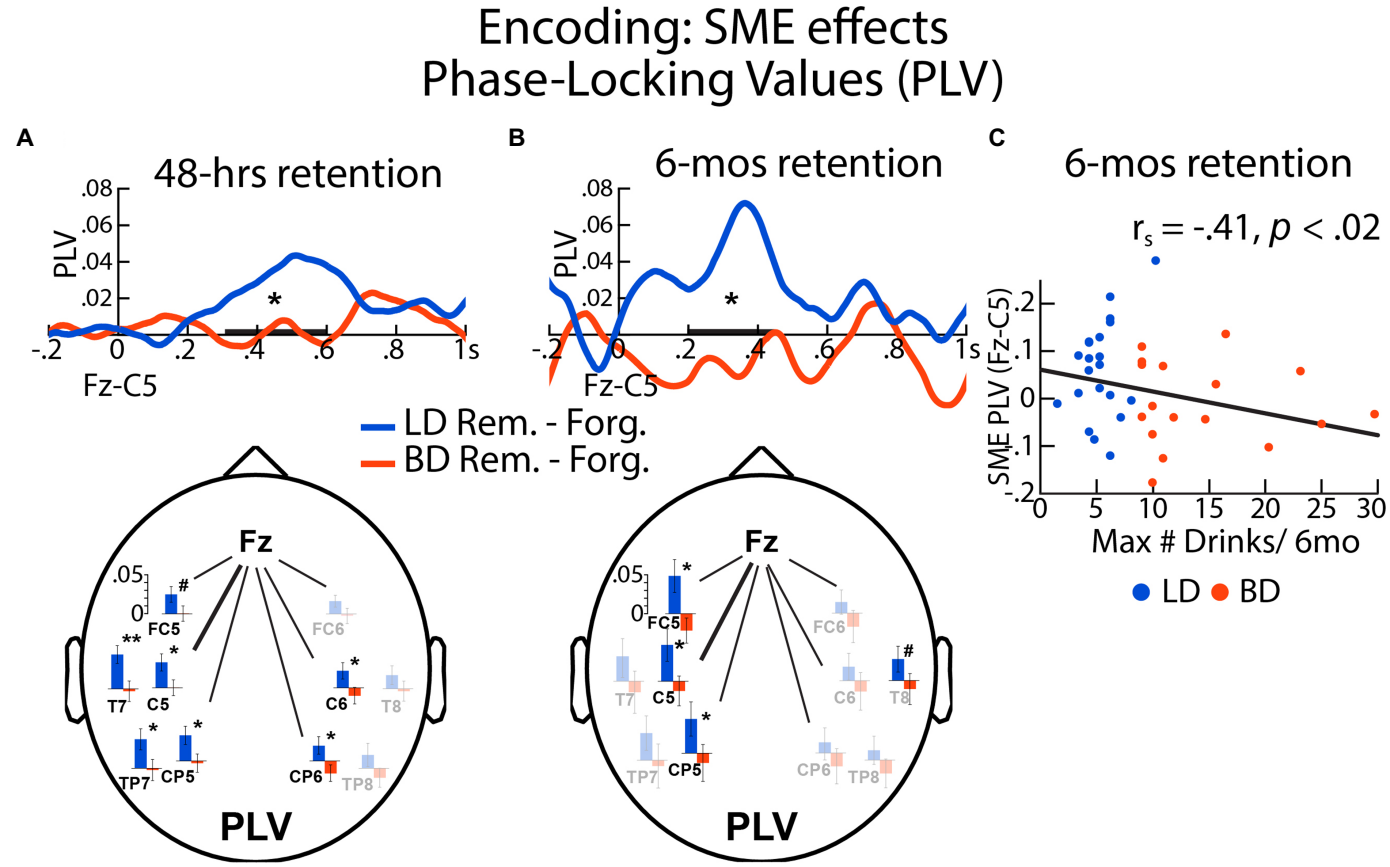
The SMEs on phase locking values (PLVs) during encoding were expressed as differences in PLVs between later remembered and later forgotten items assessed after **(A)** 48 h and **(B)** 6 mos for BD and LD groups. Here shown are the PLVs for the Fz-C5 electrode pair. **(C)** SME-evoked Fz-C5 synchrony during encoding associated with 6-mos recognition was negatively correlated with the self-reported maximum number of drinks consumed on a single occasion during the previous 6 mos. The electrode maps show the SME effects on the theta-entrained synchrony between Fz and other electrodes associated with a 48-h and a 6-mos interval, respectively. Fully colored bar graphs show the electrode pairs with significant LD – BD group differences. No group differences are marked with faint-colored bar graphs. *p < = 0.05; ^#^*p* < = 0.10; bolded bar on the x-axis represents the time window of interest.

PLVs were calculated between the frontal (Fz) and a left central (C5) electrode location to examine the oscillatory synchrony dynamics during memory encoding as a function of retention delay. For the 48-h delay, there was an interaction between Group and SME in the 300–600 ms time window, *F*(1, 64) = 4.69, *p* = 0.031. Specifically, SME (later remembered > later forgotten) was observed for LDs, *t*(32) = 2.98, *p* = 0.005, but not for BDs, *t*(32) = 0.025, *p* = 0.98. SME-related Fz-C5 PLV difference was negatively correlated with the number of reported binge episodes in the past 6 mos, *r_s_* = −0.28, *p* = 0.024, the maximum number of drinks consumed on a single occasion in the past 6 months, *r_s_* = −0.27, *p* = 0.028, enhancement drinking motives, *r_s_* = −0.32, *p* = 0.009, social drinking motives, *r_s_* = −0.26, *p* = 0.036, coping drinking motives, *r_s_* = −0.27, *p* = 0.034, and alcoholism symptoms (SMAST), *r_s_* = −0.26, *p* = 0.036, all surviving the Benjamini-Hochberg correction. While the Fz-C5 PLV time courses are shown in Figure 4 to illustrate the effect, similar effects were observed for other electrode pairs (Fz with FC5, C5, T7, CP5, TP7, C6, CP6), showing left-hemisphere dominance (Figure 4A, bottom panel, statistical comparisons are available in Supplementary Table S2 and additional timecourses are included in Supplementary Figure S1).

#### 6-mos delay

There was an interaction between Group and SME on Fz-C5 PLVs within the 200–450 ms latency, *F*(1, 35) = 5.34, *p* = 0.027, with greater neural synchrony SME in the LD compared to the BD group. In the LD group, the Fz-C5 PLV synchrony during encoding was greater for the later remembered relative to later forgotten pictures, *t*(19) = 2.53, *p* = 0.021. In contrast, there were no SME modulations of PLVs for the BD group, *t*(16) = −0.74, *p* = 0.47. Furthermore, SME-modulated Fz-C5 PLVs correlated negatively with the reported binge episodes, *r_s_* = −0.35, *p* = 0.039, the maximum number of drinks imbibed in a single occasion in the past 6 months, *r_s_* = −0.41, *p* = 0.013, and social drinking motives, *r_s_* = −0.38, *p* = 0.025. Similar effects were observed for two other left-lateralized electrode pairs (Fz-FC5 and Fz-CP5, Figure 4B bottom panel; Supplementary Table S1; Supplementary Figure S1).

## Discussion

The present study examined the SMEs on event-related theta power and phase-locked co-oscillations as a function of short (48-h) and long (6-mos) retention delays in young adult binge and light drinkers. Our findings confirm that stronger SMEs are reflected in greater event-related theta power overall. Equivalent recognition accuracy was observed in both groups after both retention delays. However, BDs showed reduced theta power during picture encoding associated with SMEs after a 6-mos retention interval. The SME correlated negatively with high-intensity drinking in the previous 6 months. In addition, only LDs but not BDs displayed SME-induced fronto-posterior theta phase synchrony in relation with both retention intervals. The SMEs for the PLVs also correlated negatively with high-intensity drinking reported for the previous 6months.

### SME-associated event-related theta power and PLVs

In the present study, the BD and LD groups differed in SMEs reflected in theta power and co-oscillations. However, we found no group differences in behavioral performance, which is broadly aligned with extant evidence. Binge drinking seems to exert a subtle impact on verbal memory performance, and only a small proportion of studies have reported impaired performance on visual memory tasks in young binge drinkers (see Carbia et al., 2018 for review; Scaife and Duka, 2009). Of note, an EEG study that demonstrated deficits in SME-associated event-related potentials (ERPs) during encoding in binge drinkers did not identify group differences in behavioral performance, either (Folgueira-Ares et al., 2017). Moreover, a recent review of EEG studies on binge drinking (Almeida-Antunes et al., 2021) reported that behavioral differences between BDs and non/low drinkers were observed in fewer than 25% of studies that employed cognitive tasks. Indeed, group differences are typically observed in studies using neural measures, often in the absence of behavioral deficits (Crego et al., 2012; Petit et al., 2014; Huang et al., 2018; Holcomb et al., 2019; Lannoy et al., 2019), suggesting the subtlety of deficits at the behavioral level. Moreover, the BD group comprised highly functional individuals whose performance did not differ from LDs on neuropsychological tests of episodic memory, working memory, processing speed, or cognitive flexibility, corroborating that EEG indices are more sensitive to neural alterations in young BDs than behavioral measures (Maurage et al., 2009; López-Caneda et al., 2017; Huang et al., 2018; Correas et al., 2019; Holcomb et al., 2019).

In the present study, SMEs were reflected in greater event-related theta power during encoding which predicted better recognition performance after a long retention delay. This finding is consistent with recent reports of increased frontal midline theta recorded during encoding of items that are subsequently remembered (see Hsieh and Ranganath, 2014 for review). Intracranial EEG (iEEG) recordings in humans indicate that theta oscillations are primarily generated in superficial cortical layers and may represent widespread integration across different cortical areas (Wang et al., 2005; Halgren et al., 2015, 2018; Solomon et al., 2019). Intracranial EEG evidence also suggests that the coherent theta-band activity in the hippocampus supports successful encoding of new items by coordinating cortical rhythmic activity (Hasselmo and Eichenbaum, 2005; Lega et al., 2012; Berens and Horner, 2017; Zheng et al., 2019). Consequently, it has been proposed that theta oscillations recorded from neocortical areas during memory formation reflect activity of the hippocampo-cortical feedback loops (Klimesch et al., 1997; Jones and Wilson, 2005; Eichenbaum, 2017). In support of this idea, our results indicate that theta activity in the neocortex, which is likely coordinated by hippocampal theta, is important for creating the integrated representations of novel items in the memory system (Siapas et al., 2005; Sauseng et al., 2007; Benchenane et al., 2010; Nyhus and Curran, 2010).

Consistent with the integrative role of theta during encoding, our PLV results indicate elevated fronto-posterior theta phase-locking during SME in the LD group. Specifically, the LD group showed greater theta-entrained PLVs between the frontal and the left-dominant posterior brain regions to the pictures that were subsequently remembered with high-confidence, relative to those that were subsequently forgotten after both retention intervals. These PLV findings align with other scalp EEG studies documenting increased theta phase synchronization between the frontal and posterior cortices during episodic memory formation (Schack and Weiss, 2005; Summerfield and Mangels, 2005; Staudigl and Hanslmayr, 2013). Such enhanced fronto-posterior theta synchrony during encoding adds to the evidence that formation of episodic memories is subserved by neural synchrony integrating diverse brain regions including the frontal and the lateral and medial temporal lobes (Paller and Wagner, 2002). Further, the stronger SMEs on theta oscillations between frontal and left posterior locations are aligned with the prior evidence of left-lateralized SME on theta oscillatory activity (Staudigl and Hanslmayr, 2013; Miller et al., 2018) and the importance of the left entorhinal cortex for successful encoding (Solomon et al., 2019). Even though the great majority of studies have probed verbal memory (Staudigl and Hanslmayr, 2013), left-lateralized theta during pictorial encoding is also sensitive to successful memory (Pu and Yu, 2019).

### Deficits in SME-associated theta oscillations in binge drinkers

While there were no group differences in SMEs on event-related theta power for a short retention interval, the SME theta modulations were attenuated in BDs when considered for the 6-mos interval. It points to selective deficits in oscillatory neural networks subserving encoding processes that dissipate over time and are not maintained over a longer delay. Similarly, the PLV data unveiled the absence of SMEs on the theta phase-locking between the frontal lobe and the posterior regions in BDs. Indeed, greater functional connectivity (Sneve et al., 2015) between the hippocampus and other areas, as well as more robust DTI connectivity with the prefrontal cortex (Cohen, 2011) result in stronger and longer lasting memory. The present finding of weaker or absent SMEs for both, event-related theta and synchronous co-oscillations in BDs compared to LDs, mirrors prior evidence of the deficient neural synchrony subserving integrative cognitive processing following acute alcohol consumption or among young binge drinkers (Beaton et al., 2018; Correas et al., 2019; Marinkovic et al., 2019).

These observations are consistent with the convergent evidence of alcohol-induced disturbances in the brain areas critical for memory formation such as the hippocampus and the prefrontal cortex (Oscar-Berman and Marinkovic, 2007; Oscar-Berman et al., 2014; Sullivan, 2017; Fama et al., 2021). Relatedly, fMRI evidence has documented atypical activation patterns in the hippocampus mediating novel encoding in teenage binge drinkers, suggesting that binge drinking may alter the neural substrate of the encoding processes in the developing brains (Schweinsburg et al., 2010). Studies in rodents have confirmed morphological changes in the hippocampus such as decreased numbers of pyramidal and dentate gyrus granule neurons (Nixon et al., 2002; Herrera et al., 2003) as well as suppressed induction of long-term potentiation (Roberto et al., 2002) following chronic exposure to ethanol. Neurodegeneration and inhibition of neurogenesis following long-term exposure to ethanol have also been documented in frontal regions in animal model studies (Crews et al., 2000; Crews and Nixon, 2009). These extensive findings from animal models provide substantial explanatory evidence for the neurophysiological underpinnings of the impairments in long-term episodic memory reported in humans with AUD (Oscar-Berman and Marinkovic, 2007; Oscar-Berman, 2012; Stavro et al., 2013).

These alterations in theta activity and fronto-posterior theta phase synchrony during encoding for long-term memory among young BDs are suggestive of a selective dysregulation of excitation/inhibition (E/I) balance that underlies the long-range co-oscillatory synchrony between the principal cortical and limbic nodes implicated in long-term memory as function of binge drinking (Klimesch et al., 2001; Siapas et al., 2005). Indeed it has been well established that neural inhibition, as effectuated by the gamma amino butyric acid (GABA), the primary inhibitory neurotransmitter, plays an essential role in stabilizing neural networks and memory consolidation (Barron, 2021). However, alcohol misuse is associated with reduced inhibitory function (Koob and Le Moal, 2008; Roberto and Varodayan, 2017). Indeed, recent evidence indicates that binge drinking is associated with lower GABA concentration (Marinkovic et al., 2022) and neural hyperexcitability (Correas et al., 2021). GABA reduction in the hippocampus is associated with neural hyperactivity and memory impairments in animal (Li et al., 2021) and human studies (Jiménez-Balado et al., 2021). Over time, alcohol-induced neurochemical changes may contribute to the allostatic neuroadaptations in limbic brain structures that are critical for memory consolidation (Koob, 2003; Koob and Le Moal, 2008; Wise and Koob, 2014). Such neuroadaptive effects observed in young BDs tip the E/I balance towards excitation, making it more difficult to encode information and retain it over longer time intervals. The altered theta-mediated memory processes may underpin the more frequent alcohol-induced blackouts that contribute to memory loss for the events occurring during intoxication (Read et al., 2013; Hingson et al., 2016), and lower academic achievement (Miller et al., 2007; Pascarella et al., 2007; Huang et al., 2018; Holcomb et al., 2019), which have been reported in college binge drinkers.

While these findings provide evidence for disrupted theta activity during memory encoding in young BDs, these deficits are broadly consistent with dysregulated theta observed in BDs during tasks probing attention (Correas et al., 2019), inhibitory control (Holcomb et al., 2019) and emotional processing (Huang et al., 2018). Furthermore, theta dysfunction during cognitive tasks has also been observed in individuals with AUD (Kamarajan et al., 2004, 2012; Jones et al., 2006) and adolescents at high risk for alcohol addiction (Porjesz and Rangaswamy, 2007). This convergent evidence suggests that theta alterations during memory processing is one aspect of a more general deficit in neurocognitive functioning in relation to binge drinking. Furthermore, binge drinking is theorized to be a transitional phase towards alcohol dependence (McCarty et al., 2004; Enoch, 2006). Therefore, these theta disturbances during mnemonic processes among BDs may be the precursor to memory impairments characterizing AUD (Oscar-Berman and Marinkovic, 2007). In addition, our results are aligned with proposals that altered theta synchrony during cognitive processes may serve as an effective neurophysiological marker of a predisposition towards the development of AUD (Porjesz et al., 2005; Rangaswamy and Porjesz, 2014). However, due to the relatively small sample size impeding the statistical power especially for the recognition assessment after a 6-mos delay, the observed group differences should be interpreted with caution. Moreover, to ensure sufficient power for the EEG analyses, trials were aggregated across emotional picture categories. Given the previous reports of altered EEG indices of emotional processing in binge drinkers (e.g., Huang et al., 2018), future studies could be designed to allow comparisons between emotional categories to examine the impact of emotional processing on SME-related EEG outcomes. Another limitation involves the potential confounding effect of volume conduction on the estimate of the theta synchronization between different electrode locations. It is recommended that future researchers endeavor to apply advanced methods (e.g., Bruña et al., 2018) to mitigate such possible effects.

### Conclusion

In conclusion, in the absence of BD vs. LD group differences in pictorial memory performance, SME theta power associated with long-term (6 mos) memory retention was attenuated in BDs compared to LDs. This observation suggests that during encoding, LDs were able to engage a distributed neural network reflected in increased theta power, supporting item retention in remote memory. In contrast, the BD group was characterized by an inefficient network-level interactive engagement of the brain areas that mediate memory formation, particularly for the items that were prospectively retained over 6 months in remote memory. This SME deficit is further substantiated by dysregulated long-range synchronous co-oscillations in BDs. The importance of excitation/inhibition (E/I) balance for the long-range corticolimbic neural synchrony that mediates memory encoding and retention is well established. At the same time, convergent animal and human mechanistic evidence indicates that E/I dysregulation is associated with alcohol misuse represented by binge drinking. Thus, the observed SEM deficits in BDs are consistent with suboptimal neural activity in the corticolimbic circuitry during encoding. Aligned with other evidence, the divergence between the behavioral and EEG results endorses the argument that direct neural measures are selectively sensitive to deficits in young BDs that are otherwise too subtle to be detected with behavioral tests (Lannoy et al., 2019; Almeida-Antunes et al., 2021). These findings address a gap in the memory literature on binge drinking and expand our current understanding of possible neural underpinnings of the early stages of alcohol use disorder. Furthermore, these results may have clinically relevant implications for the development of diagnostic and prevention strategies for problematic alcohol use by underscoring the importance of the elements that focus on memory disturbances.

## Data availability statement

The raw data supporting the conclusions of this article will be made available by the authors, without undue reservation.

## Ethics statement

The studies involving human participants were reviewed and approved by University of California, San Diego (UCSD) Human Research Protection Program (HRPP). The patients/participants provided their written informed consent to participate in this study.

## Author contributions

SH and KM designed the study. SH was responsible for collecting, analyzing, interpreting the data, and writing the manuscript. KM oversaw and contributed to all aspects of the study, including data interpretation and writing. DW assisted with data analysis and figure creation. All authors contributed to the article and approved the submitted version.

## Funding

This research was supported by start-up funds provided by San Diego State University College of Sciences and by a grant from the National Institute on Alcohol Abuse and Alcoholism (AA027371).

## Conflict of interest

The authors declare that the research was conducted in the absence of any commercial or financial relationships that could be construed as a potential conflict of interest.

## Publisher’s note

All claims expressed in this article are solely those of the authors and do not necessarily represent those of their affiliated organizations, or those of the publisher, the editors and the reviewers. Any product that may be evaluated in this article, or claim that may be made by its manufacturer, is not guaranteed or endorsed by the publisher.
