# Supplementary material for: Alterations of theta power and synchrony during encoding in young adult binge drinkers: Subsequent memory effects associated with retrieval after 48 h and 6 months
Source: Front Psychol. 2022 Dec 15;13:1061016. doi: 10.3389/fpsyg.2022.1061016 (PMC9798430; doi:10.3389/fpsyg.2022.1061016)
Supplement: Supplementary file 1 [file Data_Sheet_1.PDF]

Supplementary Table S1.

Statistical results of the SME (later remembered vs. later forgotten) × Group (LD vs. BD) ANOVA on the regional clusters of event-related theta power.

	**48-hrs retention 300-600ms**		**6-mos retention 200-450ms**	
	***F*(4, 256)**	***p***	***F*(4, 140)**	***p***
Region × SME × Group	1.57	.19	2.09	.11
	***F*(1, 64)**	***p***	***F*(1, 35)**	***p***
**Frontal cluster**				
Main effect of SME	17.50	**< .001**	10.27	**.003**
Main effect of Group	0.001	.97	0.76	.39
SME × Group	0.56	.45	9.08	**.005**
**Central cluster**				
Main effect of SME	17.86	**< .001**	8.83	**.005**
Main effect of Group	0.002	.97	1.70	.20
SME × Group	0.15	.70	6.96	**.012**
**Parietal cluster**				
Main effect of SME	12.77	**< .001**	6.58	**.015**
Main effect of Group	0.06	.82	0.97	.33
SME × Group	0.007	.94	2.92	.14
**Left Temporal cluster**				
Main effect of SME	5.29	**.025**	19.05	**< .001**
Main effect of Group	1.75	.19	0.41	.53
SME × Group	0.20	.65	0.19	.67
**Right Temporal cluster**				
Main effect of SME	8.91	**.004**	6.22	**.018**
Main effect of Group	0.03	.86	1.41	.24
SME × Group	0.96	.33	0.05	.82

Note. For SME-related theta associated with 48-hrs retention, there was no two-way or three-way interaction involving the factor of Brain Region, *p*s > .10. However, greater power of SME-induced theta was displayed over the frontal and the central regions as compared to that displayed over the left temporal region, $p = .044$ and $p = .005$, with Bonferroni correction for multiple comparisons.

For SME-related theta associated with 6-mos retention, there was no main effect of Brain Region, or any two-way or three-way interaction involving the Region factor, *p*s > .10.

These outcomes support the finding that the SME effects are not regionally specific but are subserved by distributed processes.

Supplementary Table S2.

Group comparisons in SME-modulated PLVs for all electrode location pairs for the 48-hrs and the 6-mos retention intervals:

	48-hrs retention		6-mos retention	
	t(64)	*p*	*t*(35)	*p*
Fz-C1	1.27	.21	0.41	.69
Fz-Cz	1.36	.18	0.22	.83
Fz-C2	1.74	.09	0.71	.49
Fz-FC5	1.74	.087	2.61	**.013**
Fz-C5	2.15	**.031**	2.31	**.027**
Fz-CP5	2.14	**.036**	2.04	**.049**
Fz-P7	1.37	.17	0.35	.73
Fz-T7	2.70	**.009**	1.36	.18
Fz-TP7	2.07	**.043**	1.04	.30
Fz-TP9	2.46	**.017**	0.03	.97
Fz-FC6	1.53	.13	1.57	.12
Fz-C6	2.21	**.030**	1.46	.15
Fz-CP6	2.30	**.025**	1.46	.15
Fz-P8	1.27	.21	0.13	.90
Fz-T8	1.15	.25	1.78	.084
Fz-TP8	1.51	.13	1.52	.14
Fz-TP10	0.74	.46	0.17	.87

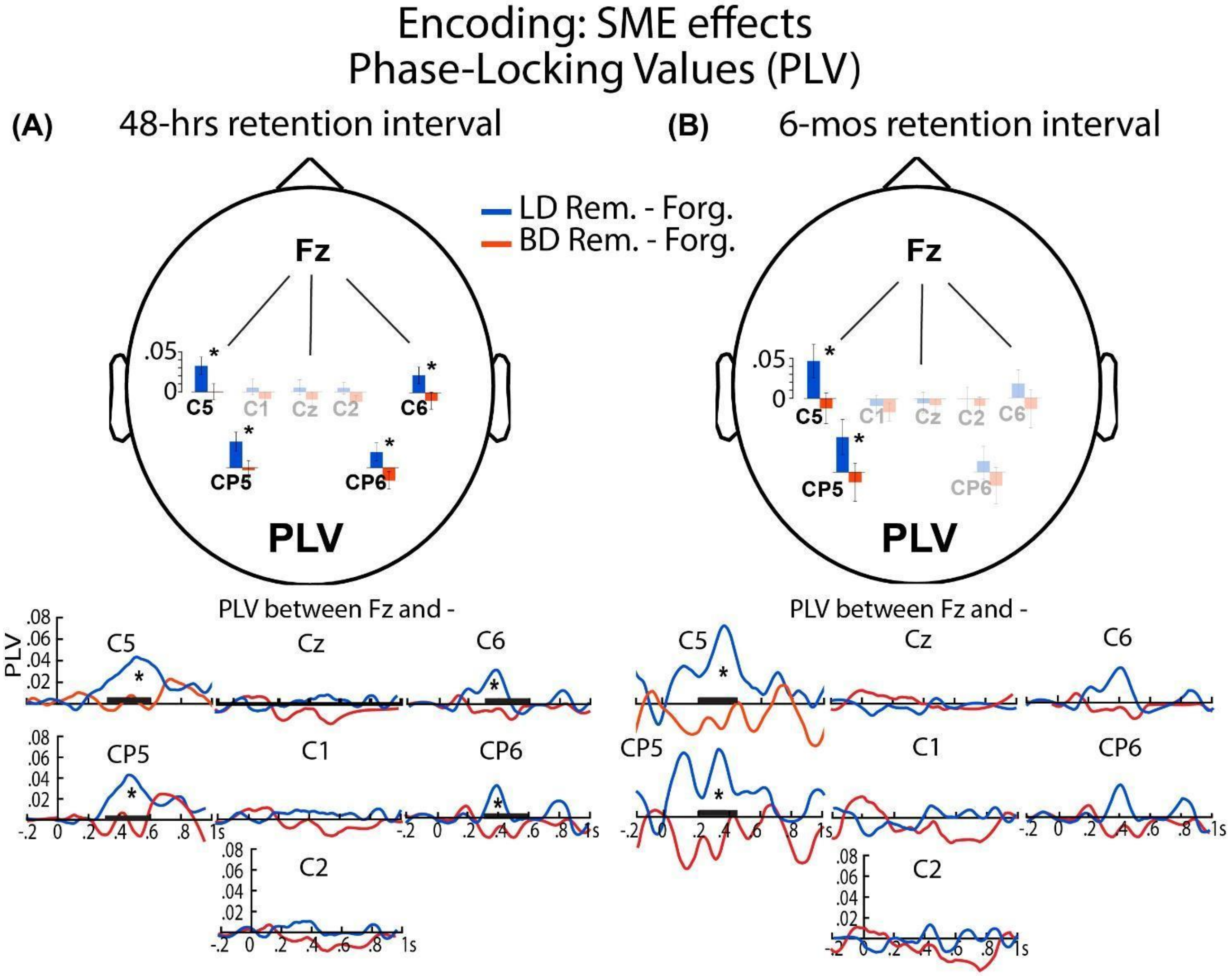

Supplementary Figure S1. The SMEs (later-remembered – later-forgotten) on PLVs between Fz and different central/central-parietal electrodes during encoding that were associated with recognition after (A) 48 hrs and (B) 6 mos. Fully colored bar graphs show the electrode pairs with significant LD – BD group differences. No group differences are marked with faint-colored bar graphs.
*p <= .05; bolded bar on the x-axis represents the time window of interest.
